# Periprocedural and 30-day outcomes of robotic-assisted percutaneous coronary intervention used in the intravascular imaging guidance

**DOI:** 10.1007/s12928-022-00864-0

**Published:** 2022-05-05

**Authors:** Yorihiko Koeda, Masaru Ishida, Koto Sasaki, Sayaka Kikuchi, Shohei Yamaya, Keiko Tsuji, Takenori Ishisone, Iwao Goto, Takumi Kimura, Yudai Shimoda, Akio Doi, Yoshihiro Morino

**Affiliations:** 1 Division of Cardiology, Department of Internal Medicine, Iwate Medical University, 2-1-1, Shiwa-gun, Yahaba-cho, Iwate 028-3695 Japan; 2 Faculty of Software and Information Science, Iwate Prefectural University, Takizawa, Japan

**Keywords:** Intravascular ultrasound, Optical coherence tomography, Optimal stenting

## Abstract

In recent years, there have been several reports on robotic-assisted percutaneous coronary intervention (R-PCI), but few studies have been conducted on R-PCI performed under intravascular imaging guidance. To elucidate the periprocedural and postoperative 30-day outcomes of intravascular imaging-guided R-PCI, we performed a retrospective observational study on all patients in 102 consecutive cases who underwent R-PCI under intravascular imaging guidance at a single center in Japan from June 12, 2019 to February 18, 2021. The primary end point was 30-day survival, and the secondary end point was the incidence of complications. Intravascular imaging-guided R-PCI was performed 110 times in total on 125 lesions. The medians of procedural time, fluoroscopy time, contrast volume, patient entrance skin dose, and radiation exposure to the main operator were 49 min, 16 min, 67 mL, 0.62 Gy, and 0 μSv, respectively. Furthermore, 60.0% of target lesion branches were American College of Cardiology Foundation/American Heart Association classification type B2 or type C. However, in all cases, lesion dilatation was successful, and the final Thrombolysis in Myocardial Infarction flow grade was 3. The combination of manual operation was required in 12.7% of all cases, but 30-day survival was confirmed in all cases. There were two problems at the puncture site. One small distal branch artery dissection occurred due to manual operation, but no cardiovascular events (myocardial infarction, stroke) occurred and no target lesion restenosis was observed within 30 days of R-PCI. Hence, R-PCI using intravascular imaging demonstrated highly satisfactory treatment outcomes, and no complication caused by robotic operation was observed.

**Supplementary Information:**

The online version contains supplementary material available at 10.1007/s12928-022-00864-0.

## Introduction

Percutaneous coronary intervention (PCI) for coronary artery disease is a non-surgical invasive procedure with the goal of relieving the narrowing or occlusion of the coronary artery and improving the blood supply to the ischemic tissue [1]. The most common method of PCI consists of ballooning the stenosis lesion or deploying a stent. The CorPath GRX System (Corindus Inc., Waltham, USA), a second-generation robotic system assisting PCI, was first clinically used in the USA, where it was developed, and it demonstrated good treatment outcomes in 40 subjects (54 lesions) [2]. Subsequently, the CorPath GRX, its successor, was introduced in Asia [3, 4] and has been used in Japan since 2019 [5, 6]. Robotic-assisted PCI (R-PCI) has the advantage of lower radiation exposure (95%) to the main operator compared to conventional manual PCI [7]. It has also been reported that precise robotic lesion length measurement contributes to appropriate stent length selection [8] [9]. CorPath GRX is also useful for tortuous and complex lesions [10], and its use in carotid artery disease has recently been reported [11, 12].

Tejas et al. performed 310 R-PCIs using the CorPath GRX from 2017 to 2019 in India and reported significantly lower radiation exposure to the patient without increasing the fluoroscopy time and contrast volume. However, the procedural time was slightly increased (5–10 min) when compared with conventional PCI [3]. In addition, in the study, with respect to 30-day major adverse cardiac or cerebrovascular events (MACCEs), there were eight (2.6%) target lesion restenosis (TLR) and one (0.3%) myocardial infarction, but there was no death and no significant difference from the conventional PCI group. On the other hand, most R-PCIs have been performed under angiography guidance, and there has been scant research on the treatment outcomes of the combination of intravascular imaging.

Intravascular imaging-guided PCI is useful for determining the position of the major axis during stenting and is said to have better treatment outcomes than angiography-guided PCI. Therefore, intravascular imaging-guided R-PCI may demonstrate treatment outcomes better than those of conventional reports. This research is an exploratory study of the treatment outcomes of intravascular imaging-guided R-PCI at a single center, and it aimed to clarify the details of the procedure, clinical outcomes, and procedural complications.

## Methods

### Study population

**Fig. 1:**
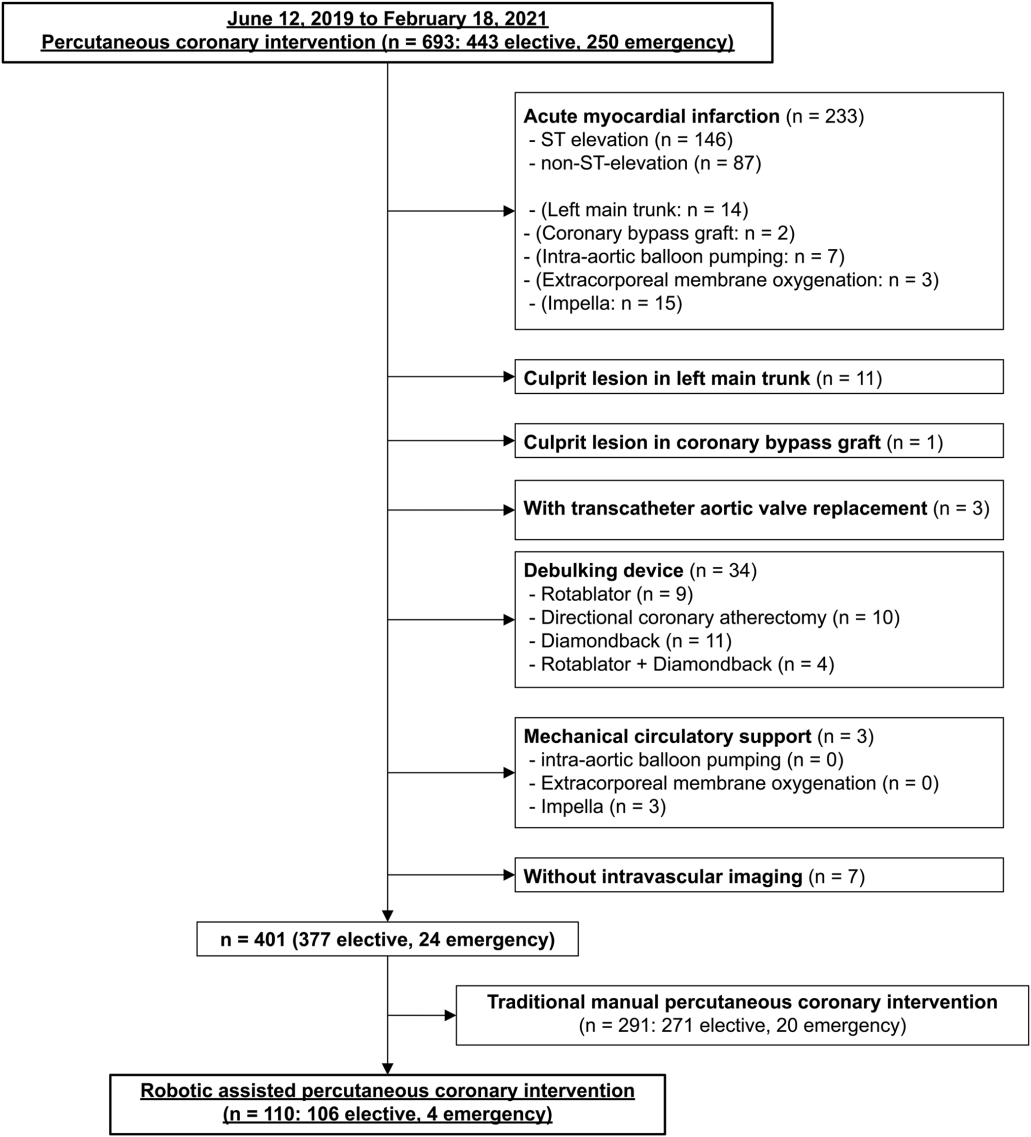
Flowchart for inclusion and exclusion criteria. Exclusion criteria were “acute myocardial infarction,” “culprit lesion in left main trunk,” “culprit lesion in coronary bypass graft,” “with transcatheter aortic valve replacement,” “using debulking device,” “using mechanical circulatory support,” and “without intravascular imaging”

The study cohort comprised all 102 consecutive patients (78 males, mean age of 70.1 ± 9.6 years, 110 PCIs, 125 lesions) who underwent R-PCI using the CorPath GRX for ischemic heart disease at Iwate Medical University Hospital from June 12, 2019 to February 18, 2021. A flowchart for inclusion and exclusion criteria are shown in (Fig. 1). Patients characterized with “Acute myocardial infarction,” “culprit lesion in left main trunk,” “culprit lesion in coronary bypass graft,” “with transcatheter aortic valve replacement,” “using debulking device,” “using mechanical circulatory support,” and “without intravascular imaging” were excluded from this study. Of the remaining 401 cases, 110 were selected for R-PCI; the selection of either R-PCI or traditional manual PCI was at the discretion of the main operator.

This study was conducted in compliance with the ethical principles of the Declaration of Helsinki (2013, Brazilian revision) and was approved by the ethics committee of Iwate Medical University (MH2021-082). Written informed consent for R-PCI was obtained from all patients, and all patients were given the opportunity to opt out from this retrospective observational study (https://iwate-heart.jp/public_information/).

With regard to the subjects’ clinical characteristics upon admission, all comorbidities and medical history were defined in accordance with the Japanese PCI (J-PCI) registry [13]. Current smoking history was defined as smoking within one year before admission. Left ventricular ejection fraction (LVEF) was measured using the Simpson method by transthoracic echocardiography. The inclusion criteria of this study were (1) stable effort angina pectoris, (2) silent myocardial ischemia, and (3) unstable angina pectoris. PCI was indicated for the stable coronary artery diseases of (1) and (2) when either of the following requirements was met: (1) ≥ 90% stenotic lesion, (2) stenotic lesion possibly causing chest symptoms, and (3) positive for functional ischemia assessments (exercise stress electrocardiogram, stress myocardial scintigraphy, fractional flow reserve, and fractional flow reserve derived from coronary CT angiography. The exclusion criteria were acute myocardial infarction, severe calcified lesion requiring debulking device, and left main trunk lesion.

### Procedures

R-PCI was operated mainly by five operators, including two Japanese Association of Cardiovascular Intervention and Therapeutics (CVIT) specialists and three CVIT-certified physicians who received training for the CorPath GRX^®^ system in advance. Meanwhile, setups, including sheath placement, guiding catheter cannulation, and single-use cassette connection, were mainly performed by the assistants.

Procedural time was defined as the time from cannulation to the removal of the guiding catheters used in R-PCI. Intraprocedural iodine contrast volume, patient entrance skin dose, fluoroscopy time, and radiation exposure to the main operator were investigated. All R-PCIs in this study were performed under intravascular imaging (intravascular ultrasound and optical coherence tomography) guidance. The CorPath GRX does not have the function of operating intravascular imaging devices; thus, these devices were used by manual operation. Intravascular imaging was performed at least twice, before and after stenting (or after planned additional dilatation). Before obtaining intravascular imaging, dilators such as isosorbide dinitrate or nicorandil were administered via coronary injection. The size and length of the stents to be implanted were determined by the operators based on the expert consensus document of the European Society of Cardiology [14]. Procedure completion was also judged by each operator based on previously established expert consensus document [14]. In this study, procedural success was defined as cases in which target stenting or balloon dilatation was successful. Procedure details of cases requiring manual operation apart from intravascular imaging (such as guide wire, device, and guiding catheter) were also investigated.

### Study end point

The primary end point was the 30-day survival after PCI, while the secondary end points were the PCI complications (myocardial infarction, coronary bypass grafting, stent thrombosis, embolism, hemorrhagic complication, aortic dissection, clear angiographical coronary dissection, pseudoaneurysm, arteriovenous fistula, and renal dysfunction) [15] and cerebrovascular events (myocardial infarction, stroke, and target lesion revascularization) within 30 days. Procedural success was defined as residual stenosis determined by quantitative coronary angiography of < 30%, and final Thrombolysis in Myocardial Infarction (TIMI) flow grade of 3. Based on the Society for Cardiovascular Angiography and Interventions (SCAI) definition, myocardial infarction as a PCI complication was defined as (1) serum creatine kinase-myocardial band (CK–MB) level (reference value 25I U/L) ≥ 10 times of the normal upper limit or myocardial troponin level ≥ 70 times of the normal upper limit or (2) serum CK–MB level ≥ 5 times of the normal upper limit or myocardial troponin level ≥ 35 times of the normal upper limit and the occurrence of a new Q wave or left bundle branch block [16]. Renal dysfunction as a PCI complication was defined as post-PCI serum creatinine level increase of ≥ 2.0 mg/dL or an increase of ≥ 50% from the previous test or new dialysis. Contrast-associated acute kidney injury (AKI) was defined as an increase in the plasma creatinine level to at least 0.5 mg/dL deciliter or at least a 25% increase from the baseline level within 2–5 days after exposure to the contrast [17].

### Statistical analysis

All values were expressed as mean ± standard deviation or median, and the Mann–Whitney *U* test was used for intergroup comparison. SPSS^®^ 25.0 for Windows (IBM, Chicago, U.S.A.) was used for statistical analysis, and *p* < 0.05 was defined as significantly different for all values.

## Results

R-PCIs comprised 15.9% (110 R-PCIs: 106 elective R-PCIs, 4 emergency R-PCIs) of the 693 PCIs (443 elective, 250 emergency) performed at our hospital during the study period. Table 1 shows the subjects’ clinical characteristics and coronary angiography findings. The mean LVEF of this cohort was good at 60.6%. However, left ventricular dysfunction with LVEF < 60% comprised 29.5% of the total, and left ventricular dysfunction with LVEF < 45% comprised 8.6% of the total. Approximately half had a history of ischemic heart disease. Among staged R-PCIs for residual stenotic lesion in patients with acute coronary syndrome who had multivessel disease (*n* = 29), 72.4% were performed within 30 days after myocardial infarction. Approximately 60% of lesions were of American College of Cardiology Foundation/American Heart Association classification type B2 or C, and chronic total occlusion (CTO) of TIMI flow grade I was confirmed in 2.7% of the total patients (*n* = 3, Case No. 46, 79, and 99).

**Table 1:** Clinical characteristics and coronary angiography findings

**Number of patients**	102		**Number of R-PCI**^a^	110	(%)
Age	70.1 ± 9.6	years			
Male	78 / 102	(76.5%)	**Clinical diagnosis**		
Body mass index	24.8 ± 4.6	kg/m^2^	Effort angina pectoris	51 / 110	(46.4)
Obesity	43 / 102	(41.2%)	Silent myocardial ischemia	26 / 110	(23.6)
Left ventricula rejection fraction	60.6 ± 9.9	%	Staged PCI^b^ for ACS patient^c^	29 / 110	(26.4)
			Unstable angina pectoris	4 / 110	(3.6)
			**Therapeutic adaptation**		
**Medical history**			> = 90% stenosis	78 / 110	(70.9)
Hypertension	93 / 102	(91.2%)	Exercise stress electrocardiogram	7 / 110	(6.4)
Diabetes mellitus	49 / 102	(48.0%)			
Dyslipidemia	96 / 102	(94.1%)	FFR^d^ < 0.80	20 / 110	(18.2)
Current smoking	15 / 102	(14.7%)	FFR-CT^†^ < 0.80	3 / 110	(2.7)
Ischemic heart disease	52 / 102	(51.0%)	Hypoperfusion in stress myocardial perfusion image	2 / 110	(1.8)
Stroke	10 / 102	(9.8%)			
Atrial fibrillation	10 / 102	(9.8%)			
Renal dysfunction	24 / 102	(23.5%)	**Culprit lesion branch**		
Hemodialysis	2 / 102	(2.0%)	Left main trunk	0	
			Left anterior descending branch	54 / 110	(49.1)
**Laboratory data**			Left circumflex branch	31 / 110	(28.2)
Estimated glomerular filtration rate	65.0 ± 17.3	mL/min/1.73m^2^	Right coronary artery	24 / 110	(21.8)
			Double vessel	1 / 110	(0.9)
Total cholesterol	154.7 ± 35.8	mg/dL			
High-density lipoprotein cholesterol	49.2 ± 13.6	mg/dL	**Bifurcation lesion**	40 / 110	(36.4)
			**Number of lesion branch (1/2/3)**	87 / 17 / 6	(79.1/15.5/5.5%)
Low-density lipoprotein cholesterol	88.5 ± 33.7	mg/dL	**Pre-interventiona**l **TIMII**^††^**flow grade ( 0 / I / II / III)**	0 / 3 / 8 / 99	
				(0 / 2.7% / 7.3% / 90.0%)	
Triglyceride	131.4 ± 93.1	mg/dL	**ACC/AHA**^††^**classification of coronaryLesions (A/B1/B2/C)**	12 / 32 / 31 / 35	
Hemoglobin A1c	6.6 ± 1.2	%		(10.9% / 29.1% / 28.2% / 31.8%)	

^a^Robotic-assisted percutaneous coronary intervention

^b^Percutaneous coronary intervention

^c^Acute coronary syndrome

^d^Fractional flow reserve

^†^Computed tomography

^††^Thrombolysis in Myocardial Infarction

^†††^American College of Cardiology/American Heart Association

Post-R-PCI 30-day follow-up rate was 100%, and there was no death, cerebrovascular event, or TLR (Table 2). In addition, in this cohort, despite mild to moderate complications, such as subcutaneous bleeding at the puncture site, distal branch coronary artery dissection due to the manual operation of the guide wires, femoral artery pseudoaneurysm, and contrast-associated AKI, there was no procedural complication caused by robotic operation.

**Table 2:** Complications and clinical outcomes of robotic-assisted percutaneous coronary intervention

30 days outcome		
All-cause death	0 / 110	
Cardiovascular event	0 / 110	
Target vessel revascularization	0 / 110	

Procedural complications		Details
Death	0 / 110	
Myocardial infarction	0 / 110	
Coronary bypass grafting	0 / 110	
Thromboembolism	0/ 110	
Hemorrhagic complication	1 / 110 (0.9%)	No. 41: a 78-year-old female. Subcutaneous hematoma by the guide wire for sheath in right radial artery (hemoglobin 12.5 → 9.8 mg/dL, no blood transfusion). R-PCI^a^ was performed via the left radial artery approach
Coronary artery dissection	1 / 110 (0.9%)	No. 77: an 80 year-old-male. R-PCI^a^ was performed successfully. The guide wire dislodged during IVUS^b^, wire crossed by manual operation. A coronary artery dissection was formed in a distal side branch. There was no chest pain, ST-segment elevation, or elevation of myocardial deviating enzymes
Artery dissection (puncture site)	0 / 110	
Pseudoaneurysm (puncture site)	1 / 110 (0.9%)	No. 33: a 66-year-old male, who had a 15-year history of dialysis, hypertension, and diabetes mellitus. R-PCI^a^ was performed via the right femoral artery approach. Although he was discharged the next day, a pseudoaneurysm at the puncture site was found after 3 days (hemoglobin 10.7 → 9.4 mg/dL, no blood transfusion). Surgical repair was performed after 4 days (packed red blood cells 2 units transfusion). He was discharged again after 10 days
Arteriovenous fistula (puncture site)	0 / 110	
Contrast-associated acute kidney injury (AKI)	5 / 110 (4.5%)	No. 7: a 66-year-old male. Serum creatinine level transiently increased from 0.68 mg/dL to 0.88 mg/dL after exposure contrast volume 91 mL
		No. 22: an 83-year-old female. Serum creatinine level transiently increased from 0.71 mg/dL to 1.09 mg/dL after exposure contrast volume 82 mL
		No. 24: a 72-year-old male. Serum creatinine level transiently increased from 0.84 mg/dL to 1.16 mg/dL after exposure contrast volume 59 mL
		No. 82: a 66-year-old male. Serum creatinine level transiently increased from 0.62 mg/dL to 0.79 mg/dL after exposure contrast volume 42 mL
		No. 110: an 80-year-old male. Serum creatinine level transiently increased from 1.20 mg/dL to 1.61 mg/dL after exposure contrast volume 86 mL

Others		Details
Coronary slow-flow phenomenon (transient)	3 / 110 (2.7%)	No. 7: a 66-year-old male. Transient coronary slow flow occurred at the guide wire crossing a side branch. He had a chest pain and ST-segment elevation, which had improved post-dilatation. The peak CK/CK–MB^c^ level was 370/31 IU/L
		No. 25: an 81-year-old female. Transient coronary slow flow in a side branch after stent dilatation. She had no chest pain and ST-segment elevation. The peak CK/CK–MB^c^ level was 347/37 IU/L
		No. 34: a 79-year-old male. Transient coronary slow flow in the main branch after stent dilatation. He had a chest pain and ST-segment elevation, which had improved at the end of R-PCI. The peak CK/CK–MB^c^ level was 356/32 IU/L

^a^Robotic-assisted percutaneous coronary intervention

^b^Intravascular ultrasound

^c^Creatine kinase

**Fig. 2:**
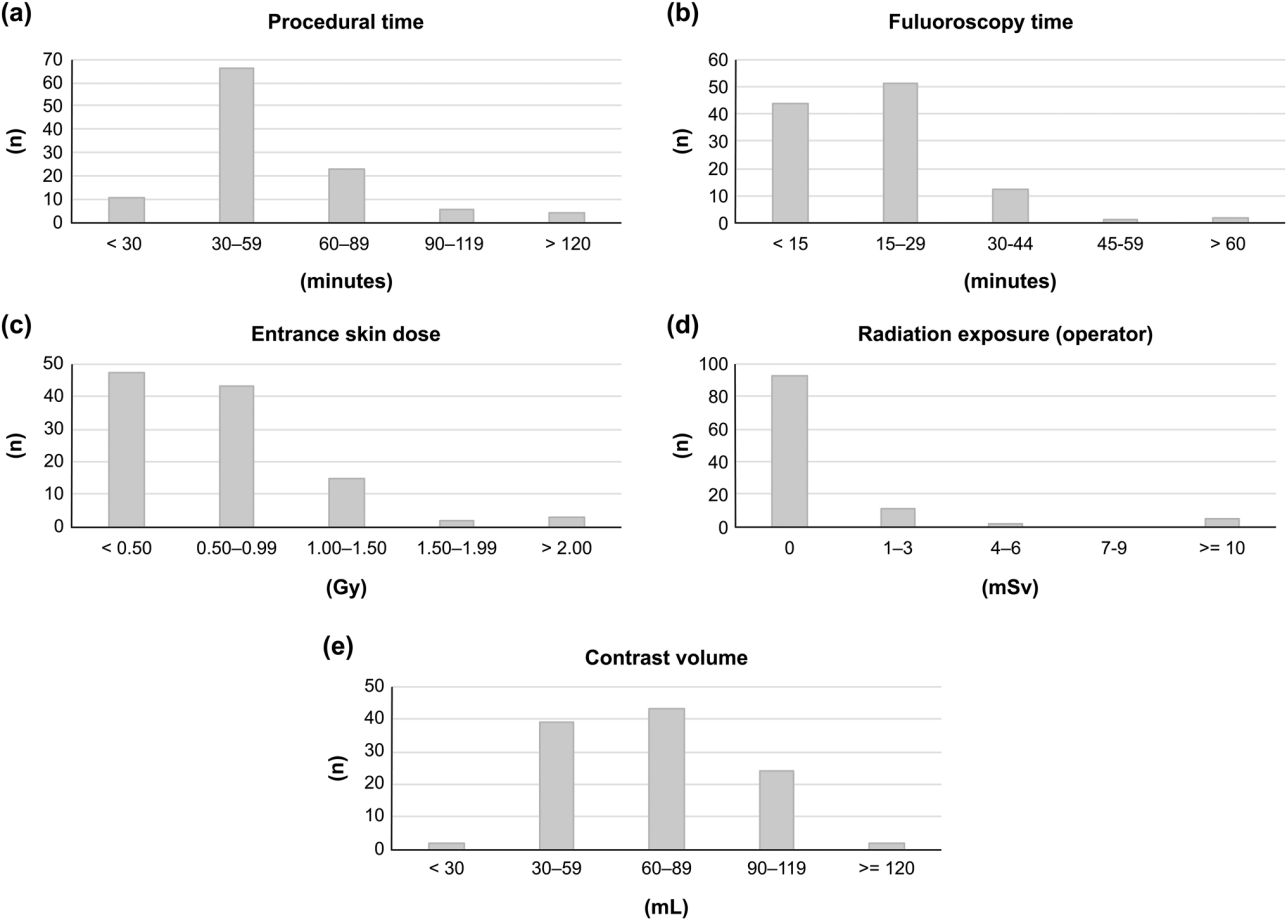
Graphs of each factor. Graphs of each factor in (Table 2). It was observed that 77.3% of all R-PCIs had a procedural time of < 60 min, 86.4% had a fluoroscopy time of < 30 min, 81.8% had an entrance skin dose of < 1.00 Gy, 83.6% had 0 μSv radiation exposure to the main operator, and 86.4% had an iodine contrast volume of < 100 mL

Table 3 and Fig. 2 show the results of the R-PCI procedure. Intravascular imaging devices were used in all cases. In 41.5% of the cases, additional balloon dilatation was performed in the implanted stents. No case required unplanned additional stenting because of coronary artery dissection. Procedural success for lesions was achieved in all 110 cases that underwent R-PCI. Most R-PCIs had a procedural time of 30–90 min, while cases completed in < 30 min and those requiring > 90 min were approximately 10% each. Based on the graph, procedural time tended to be long in the 13 cases at the early stage of introducing R-PCI, but it reduced significantly afterward (*p* = 0.003, Mann–Whitney *U* test, Supplemental Fig. 1). Furthermore, 86.4% of all R-PCIs had a fluoroscopy time of < 30 min, 81.8% had an entrance skin dose of < 1.00 Gy, 83.6% had 0 μSv radiation exposure to the main operator, and 86.4% required iodine contrast volumes within 100 mL. Most of the intravascular imaging modalities used in this study were intravascular ultra sound. Optical coherence tomography was used for patients with severe calcification in the culprit lesion or in-stent restenosis. Intravascular imaging was performed before first ballooning in about half of the cases, and post-stenting in all cases. Touch-up ballooning was added after intravascular imaging in about 40% of post-stenting cases. On the other hand, there was no case that required additional stenting. In all cases, final TIMI flow grade was 3, and percent diameter stenosis after R-PCI was under 30%.

**Table 3:** Result of robotic-assisted percutaneous coronary intervention

**Number of R-PCI**^a^	110		**Intravascular imaging device**		
**Number of treated lesions**	125		−Intravascular ultra sound (IVUS)		
			(1) AltaView^®^ / VISICUBE^®^		73 / 110 (66.4%)
**Access site**		(Total *n* = 110)	(2) OptiCross™ / iLab™		31 / 110 (28.2%)
–Radial (right /left)	72 / 30	(92.7%)	–Optical coherence tomography (OCT)		
–Distal radial (right/left)	0 / 4	(3.6%)	(3) ILUMIEN™ / OPTIS™		3 / 110 (2.7%)
–Brachial (right/left)	1 / 0	(0.9%)	–Optical frequency domain imaging (OFDI)		
–Femoral (right/left)	3 / 0	(2.7%)	(4) FastView®/LUNAWAVE®		2 / 110 (1.8%)
			–Composite (IVUS + OCT)		
**Procedural time (median)**	49.0	Minutes	(1) + (3)		1 / 110 (0.9%)
**Fluoroscopy time (median)**	16.0	Minutes			
**Entrance skin dose (median)**	0.62	Gy	**Number of drug- eluting stent**s	129	
**Contrast volume (median)**	67.0	mL	–Synergy^®^	47 / 129	(36.4%)
**Radiation exposure to the** **main operator (median)**	0	μSv	–Orsiro^®^	43 / 129	(33.3%)
			–Ultimaster TANSEI^®^	29 / 129	(22.5%)
**Radiation exposure to the** **assistant (median)**	23.5	μSv	–Xience Sierra^®^	7 / 129	(5.4%)
			–Xience Xpedition^®^	3 / 129	(2.3%)
**Drug-eluting stent**	106 / 110	(96.4%)	**Drug-eluting stent diameter**		
**Direct stenting only**	21 / 110	(19.1%)	− 2.25 mm	14 / 129	(10.9%)
**Drug-coated balloon**	5 / 110	(4.5%)	− 2.5 mm	30 / 129	(23.3%)
**Debulking device**	0		− 2.75 mm	9 / 129	(7.0%)
			− 3.0 mm	36 / 129	(27.9%)
**Procedure of intravascular imaging**			− 3.5 mm	27 / 129	(20.9%)
−Pre-ballooning	62 / 110	(56.4%)	− 4.0 mm	13 / 129	(10.1%)
−Pre-stenting	105 / 106	(99.1%)	**Drug-eluting stent length**	26.9 ± 9.2	mm
−Post-stenting	106 / 106	(100%)	(Median)	28.0	mm
−Number of imaging use	2.7 ± 0.8				
			**Post % diameter stenosis (%DS)**	7.8 ± 2.4	%
**Additional procedure after intravascular imaging (post-stenting)**			(Median)	8.4	%
−Unplanned ballooning (touch-up)	44 / 106	(41.5%)	**Post %DS < 30%**	100	%
−Additional stenting	0				
			**Minimum stent area**	6.1 ± 2.8	mm^2^
**Post-interventional** **TIMI **^b^**flow grade = III**	100	%	(Median)	5.6	mm^2^
			**Switching manual operation**	14 / 110	(12.7%)

^a^Robotic-assisted percutaneous coronary intervention

^b^Thrombolysis in Myocardial Infarction

**Fig. 3:**
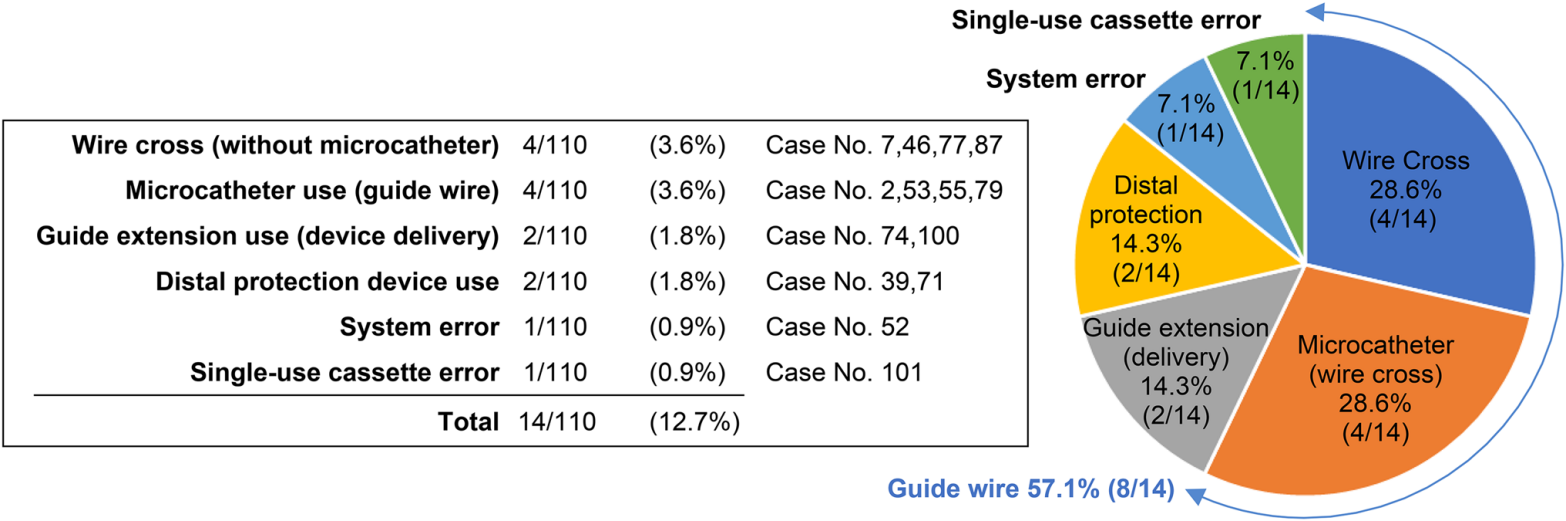
Major causes and details of manual operations besides intravascular imaging device. Manual operations used in combination in addition to intravascular imaging were mostly related to guide wire operations. System error was recovered without any issue following rebooting after R-PCI was completed. Single-use cassette failure was solved after cleaning the connection part of the robotic drive of the adhered contrast agent and rebooting

Manual operation was used in combination in 14 cases (Fig. 3). The procedural time of the robotic operation only group was significantly shorter than that of the manual operation combination group (median 43.5 min vs. 77.5 min, *p* < 0.001, Mann–Whitney *U* test, Supplemental Fig. 1). With respect to the details of manual operations, guide wire operation, microcatheters, and distal protection devices were often used for difficult lesions. The switch to manual operation was due to system error in Case No. 52 and single-use cassette error in Case No. 101, but the CorPath GRX operated without any issue after rebooting.

## Discussion

This study is the first report on detailed real-world data of intravascular imaging-guided R-PCI performed on > 100 patients. The procedure was safely executed in all cases, the 30-day outcomes were satisfactory, and there were few procedural complications. Based on the results of this study, R-PCI using intravascular imaging could be safe and feasible.

In spite of fluoroscopy and procedural time in this study were longer compared to a previous study [3], the results of the 30-day MACCEs were excellent. Although it is estimated that this difference in outcomes could largely be due to the difference in population, optimal stenting by using intravascular imaging might have contributed to the favorable prognosis [18, 19]. In this cohort, unplanned additional stenting was unnecessary because of the implantation of appropriately sized stents at the appropriate area of culprit lesions. Moreover, there was no 30-day TLR possibly because appropriate balloon dilatation was added by post-stenting intravascular imaging.

In this study cohort, an experience of 13 cases was required to achieve a short R-PCI procedural time. Later, the procedural time was often extended because of the combination of manual operation, challenging cases, or the participation of new operators; nevertheless, the procedural time was generally stable. Therefore, it was estimated that 10–15 cases were required for multiple operators at a single center to become familiarized with the operation of R-PCI, including the operation of intravascular imaging devices via single-use cassettes.

The combination rate of manual operation in this study was similar to that of previous reports [6, 20], and the use of imaging catheter devices did not increase the number of cases that required switching to other manual operations. Most manual operations used in combination involved guide wires or microcatheters. Another center has also reported that the majority of manual operations consisted of microcatheter use [6]. The “technIQ™ Smart Procedural Automation” has been recently developed as a promising new set of automated robotic movements for the CorPath^®^ GRX system for guide wire operation and device delivery. In addition, opportunities for R-PCI utilization will be further expanded when intravascular imaging device operation by robotic arms as well as microcatheter and guide extension catheter use becomes possible in the future.

Based on the good treatment outcomes, we have some expectations for the future of R-PCI, namely R-PCI for complex lesions, such as CTO and severe calcification, and remote PCI. With respect to CTO-PCI, the partially robotic-assisted group had total surgery time, contrast volume, and MACCE frequency equivalent to those of the totally manual PCI group [21]. As CTO-PCI has a long procedural time, the continuation of procedure using robotic assistance after manually wire crossing the lesions by guide wires under microcatheters is a logical strategy to reduce the physical burden and radiation exposure of the operator. Intravascular imaging is effective in PCI for CTO [22]. Good outcomes were achieved in the three cases of CTO that underwent PCI in this study, and we hope to further utilize R-PCI and accumulate more findings in the future. It has been reported that remote PCI has already successfully treated five cases in India [23]. The ability to use intravascular imaging guidance in remote PCI may lead to improvement in the treatment outcomes. Furthermore, the introduction of unprecedented novel technologies, such as the development of imaging modalities that can be combined with R-PCI and the system where artificial intelligence semi-automatically performs stenting based on imaging analysis results, is anticipated.

### Study limitations

This study has several limitations. First, this study is a single-center study, and it is possible that subject selection bias might have affected the results. However, the completion of the treatment for > 100 patients without major complications demonstrates the effectiveness of intravascular imaging-guided R-PCI. Second, this cohort is a single-group retrospective observational study of limited cases. Hence, the number of cases and the follow-up period are still insufficient for a precise comparison with conventional manual PCI using intravascular imaging guidance. To overcome this limitation, it is necessary to investigate it further in a large-scale randomized controlled trial. In the future, we plan to accumulate more procedural experience and continue following up on the study cohort.

In conclusion, intravascular imaging-guided R-PCI showed good initial treatment outcomes, and at present, is being safely executed without complications due to robotic maneuver.

## Supplementary Information

Below is the link to the electronic supplementary material.

Supplementary file1 (DOCX 273 KB)
