# Supplementary material for: Periprocedural and 30-day outcomes of robotic-assisted percutaneous coronary intervention used in the intravascular imaging guidance
Source: Cardiovasc Interv Ther. 2022 May 5;38(1):39–48. doi: 10.1007/s12928-022-00864-0 (PMC9810557; doi:10.1007/s12928-022-00864-0)
Supplement: Supplementary file 1 — Supplementary file1 (DOCX 273 KB) [file 12928_2022_864_MOESM1_ESM.docx]

**Periprocedural and 30-day outcomes of** **robotic-assisted percutaneous coronary intervention used in the** **intravascular imaging guidance**

**First author:** Koeda Y

Running title: Study of intravascular imaging-guided robotic-PCI

Yorihiko Koeda^1)^, Masaru Ishida^1)^, Koto Sasaki^1)^, Sayaka Kikuchi^1)^

Shohei Yamaya^1)^, Keiko Tsuji^1)^, Takenori Ishisone^1)^, Iwao Goto^1)^

Takumi Kimura^1)^, Yudai Shimoda^1)^, Akio Doi^2)^, Yoshihiro Morino^1)^

1. Division of Cardiology, Department of Internal Medicine, Iwate Medical University
2. Faculty of Software and Information Science, Iwate Prefectural University

**Corresponding author:** Yorihiko Koeda, M.D., Ph.D.

Division of Cardiology, Iwate Medical University, 2-1-1, Yahaba-cho, Shiwa-gun, Iwate 028-3695, Japan

**Tel:** +81-19-613-7111, Fax: +81-19-907-7279

**Email:** [yrkoeda@iwate-med.ac.jp](mailto:yrkoeda@iwate-med.ac.jp)

**
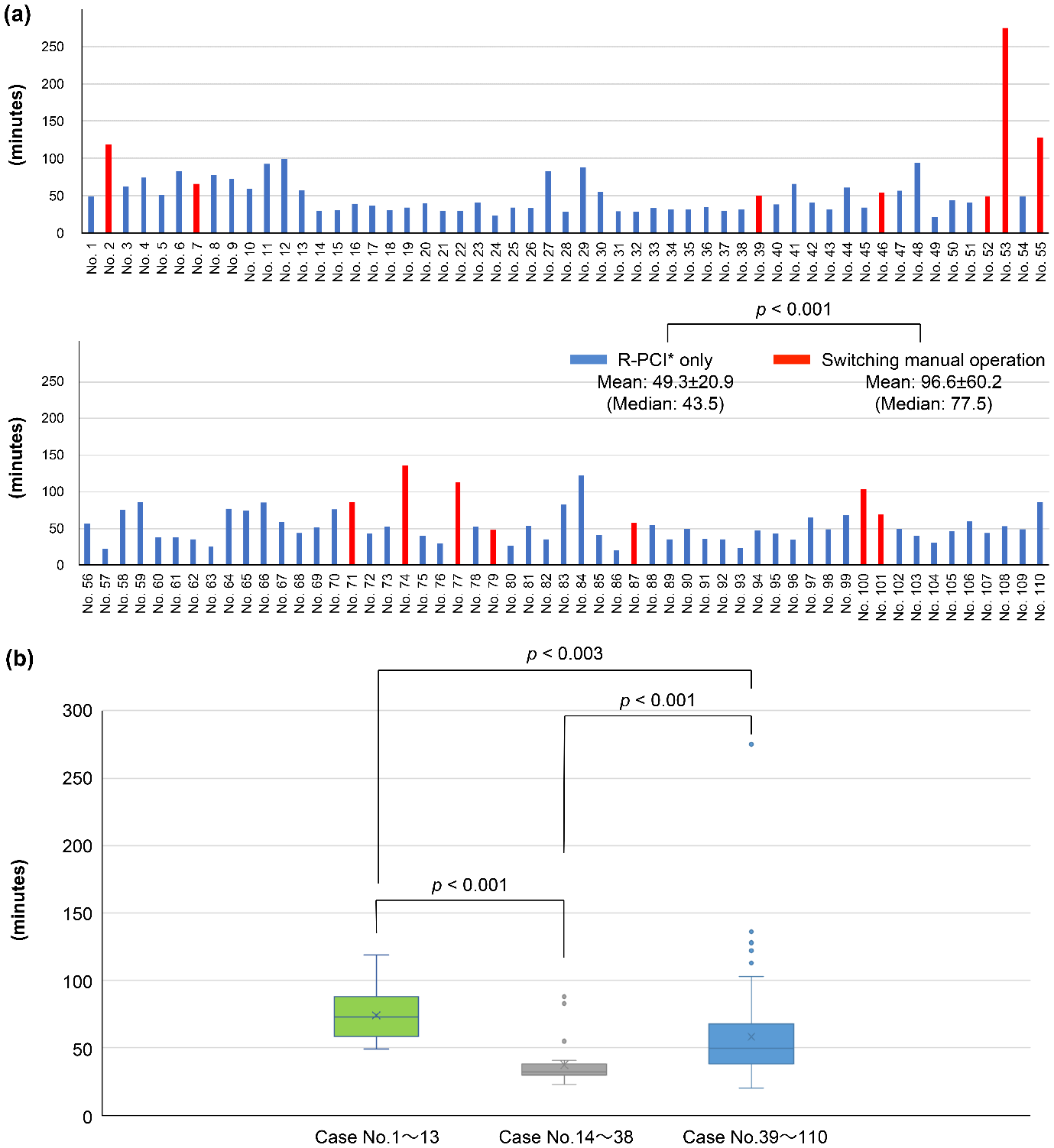
**

**Supplemental Figure 1.** **Procedural time and case number**

**(a)** Details of procedural time in Table 2 and Figure 1a. The procedural time of the manual operation combination group was significantly longer than that of the robotic operation only group (median 43.5 minutes vs. 77.5 minutes, *p* < 0.001). **(b)** Box plot of procedural time. The median procedural time of the initial 13 cases was 73 minutes, 32 minutes for the mid-term 25 cases with stable procedure, and 49.5 minutes for the late 72 cases, including challenging cases for complex lesions.

*robotic-assisted percutaneous coronary intervention.
